# Cisplatin-based chemoradiation decreases telomerase-specific CD4 TH1 response but increases immune suppressive cells in peripheral blood

**DOI:** 10.1186/s12865-021-00429-5

**Published:** 2021-06-18

**Authors:** Jihane Boustani, Elodie Lauret Marie Joseph, Etienne Martin, Salim Benhmida, Benoit Lecoester, Florent Tochet, Céline Mirjolet, Cédric Chevalier, David Thibouw, Noémie Vulquin, Stéphanie Servagi, Xushan Sun, Olivier Adotévi

**Affiliations:** 1 Department of Radiation Oncology, University Hospital of Besançon, 25000 Besançon, France; 2 INSERM, EFS BFC, UMR1098, RIGHT, Interactions Greffon-Hôte-Tumeur/Ingénierie Cellulaire et Génique, University of Bourgogne Franche-Comté, 25000 Besançon, France; 3 Department of Radiation Oncology, Centre George François Leclerc, 21079 Dijon, France; 4 INSERM UMR 1231, 21079 Dijon, France; 5 Department of Radiation Oncology, Institut Godinot, 51100 Reims, France; 6 Department of Radiation Oncology, North Franche-Comté Hospital, 25200 Montbéliard, France; 7 Department of Medical Oncology, University Hospital of Besançon, 25000 Besançon, France

**Keywords:** Chemoradiation, Immune suppressive cells, Tumor-specific T cell response

## Abstract

**Background:**

The synergistic effect of chemoradiation (CRT) has been previously demonstrated in several cancer types. Here, we investigated the systemic immune effects of CRT in patients with lung or head and neck cancer.

**Materials and methods:**

Peripheral blood mononuclear cells were collected at baseline and 1 month after treatment from blood samples of 29 patients treated with cisplatin-based chemoradiotherapy for lung or head and neck cancer. Circulating anti-tumor Th1 response was assessed by the ELISpot assay using a mixture of human leucocyte antigen (HLA) class II restricted peptides derived from telomerase (TERT). Phenotyping of circulating immunosuppressive cells (Treg and MDSC) was performed by flow cytometry.

**Results:**

A significant increase of circulating Treg was observed in 60% of patients after CRT The mean rate of Treg was 3.1% versus 4.9% at baseline and after CRT respectively, *p* = 0.0015). However, there was a no significant increase of MDSC rate after CRT. In contrast, a decrease of tumor-specific Th1 response was documented in 7 out of 10 evaluated patients. We found high frequency of pre-existing tumor-specific Th1 response among patients with objective response after CRT compared to non-responders.

**Conclusion:**

Cisplatin-based CRT promotes expansion of Treg and decrease of circulating anti-tumor Th1 response in peripheral blood. The balance towards a sustained specific anti-tumor T-cell response appears to be associated with response to CRT.

**Supplementary Information:**

The online version contains supplementary material available at 10.1186/s12865-021-00429-5.

## Background

Concurrent chemoradiation (CRT) represents a standard curative treatment for several locally advanced cancers [1]. The addition of chemotherapy (CT) to radiation therapy (RT) improves locoregional control via a synergistic effect through the induction of irreversible DNA damages [1]. During the past decade, major findings have described the immunological effects of cytotoxic anticancer therapy. Indeed, CT and RT used as monotherapy exert their anti-tumor effect not only directly by creating DNA lesions that lead eventually to cell killing, but also indirectly by stimulating an anti-tumor immune response via the innate and adaptive immunity [2–4]. Tumor cells exposed to RT and/or CT release tumor-associated antigens (TAA) which are captured by dendritic cells (DCs) for processing and presentation on MHC class I and II molecules to T cells [5]. This leads to the priming and activation of effector T-cell responses against the TAA. The activated effector T cells traffic to tumor site where they specifically recognize and kill their target cancer cells. Also, RT may cause regression of tumors distant from the irradiated site, a phenomenon known as abscopal effect. Despite being rarely observed in daily practice, preclinical and clinical evidence have suggested that this effect may be immune-mediated, translating the systemic anti-tumor effect of local RT. [6–8] On the other hand, RT was shown to drive the accumulation of immunosuppressive cells such as regulatory T cells (Tregs), myeloid-derived suppressor cells (MDSCs) and type 2 macrophages in the tumor micro microenvironment [9–12]. Furthermore RT can induce PD-L1 expression on both tumor cells and immune cells as well as upregulation of immune checkpoint receptors (TIGIT, TIM3 …) on tumor infiltrative lymphocytes, hence limiting the anti-tumor immunity [13–17].

Although the effect of each of RT and CT on immune response has been widely studied, little is known about the impact of their combination on the immune system [18, 19]. Understanding the immune modulatory properties of concurrent CRT has gained a great interest in the field of the combination with cancer immunotherapy [20–22].

CD4 T cells play a central role in orchestrating the adaptive immune response [23]. They can kill tumor cells that express MHC-II molecules either directly via MHC-II/peptide recognition [24] or indirectly by inducing MHC-II expression via IFN-γ [25–27]. Since MHC-II peptides have a lower MHC binding affinity than MHC-I peptides [28–30], CD4 T cells could have a wider range of regulation of the antitumor response. Telomerase reverse transcriptase (TERT) is a self-tumor antigen that plays a major role in tumor development and progression [31, 32], and is overexpressed in more than 90% of human tumors. Naturally occurring HLA-II-restricted CD4 T cell responses against TERT peptides were detected in patients with various types of cancer and were associated with a good prognosis [33–35]. Thus, the assessment of anti-TERT CD4 Th1 cell immunity in circulating lymphocytes has been used as a tool for monitoring antitumor CD4 Th1 response in cancer patients [33–35].

In this study, we assessed the effect of concurrent CRT on peripheral tumor-specific CD4 Th1 response and immunosuppressive cells in patients with lung or head and neck cancer.

## Results

### Patients’ characteristics

Between September 2014 and December 2016, 26 patients with lung cancer and 3 patients with head and neck cancer were included. Patients’ characteristics are shown in Table 1. All patients had locally advanced inoperable disease with CRT indication. The median radiation dose was 66 Gy (range, 34–70 Gy). Patients with small-cell lung cancer (*n* = 3) received 45 Gy. Patients with non-small cell lung cancer received 60 to 66 Gy with doses under 60 Gy in 3 patients only. Head and neck cancer patients received 70 Gy. A platinum-based concurrent CT was associated.

**Table 1:** Patients’ characteristics

		***N*** = 29	
**Age (years)**	Mean	64	
	Median (range)	65	(39–81)
**Gender n (%)**	Male	19	(65.5)
	Female	10	(34.5)
**Performance status n (%)**	0	13	(44.8)
	1	16	(55.2)
**Histologic subtype n (%)**	Adenocarcinoma	9	(31.0)
	Squamous cell	17	(58.6)
	Neuro-endocrine	3	(10.4)
**Stage n (%)**	II	4	(13.8)
	III	21	(72.4)
	IV	4	(13.8)
**Chemotherapy n (%)**	Platinum doublet	26	(89.6)
	Monotherapy	3	(10.4)
**Radiation dose (gray)**	Mean	61.6	
	Median (range)	66	(37–70)

### Increase of circulating immunosuppressive cells after CRT

**Fig. 1:**
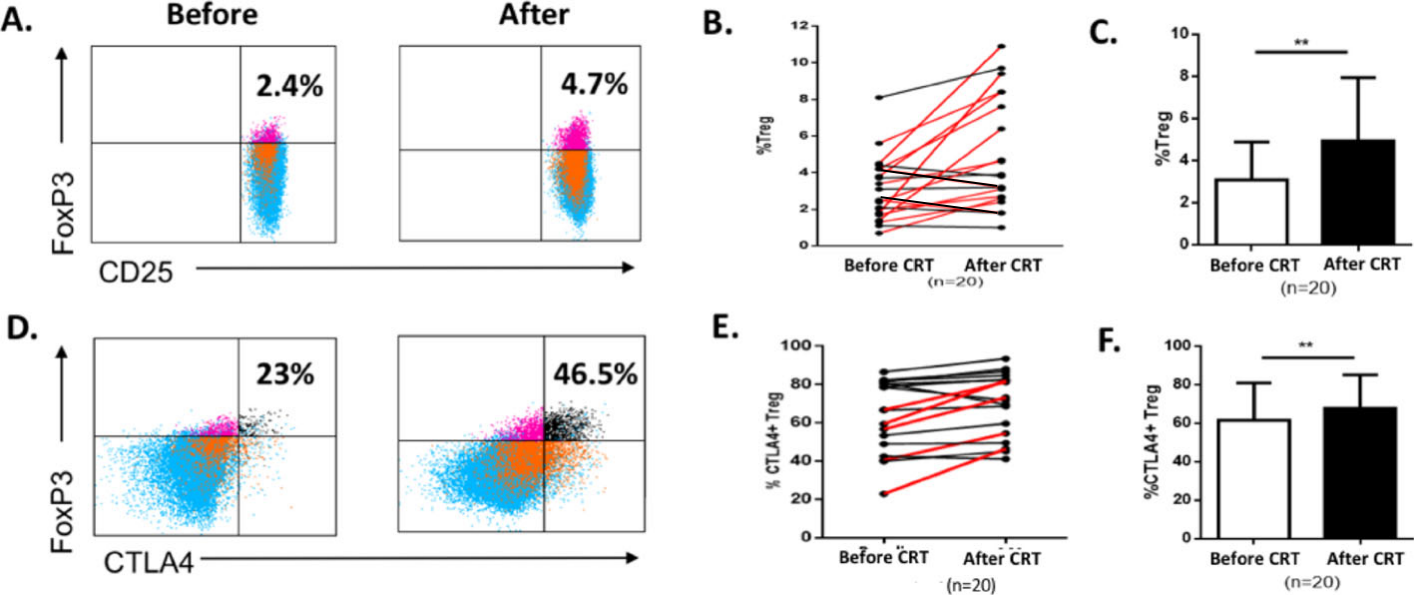
Circulating Treg cells before and after CRT. PBMCs from 20 patients treated with CRT for lung or head and neck cancer were taken before CRT and 1 month after. CD4^+^CD25^+^CD127^low^FoxP3^+^Treg were analyzed by flow cytometry. **A** and **D** Representative plots for Treg (**A**) and CTLA-4^+^ Treg (**D**) in one patient. **B** and **E** Treg (**B**) and CTLA4^+^ Treg (**E**) rates variation after CRT, lines in red representing significant increase (> 20%) from baseline (*n* = 20). **C** and **F** Treg (**C**) and CTLA-4^+^ Treg (**F**) rates before and after CRT (*n* = 20). Results are shown as mean (standard deviation). **, *p* < 0.005 (Wilcoxon test)

**Fig. 2:**
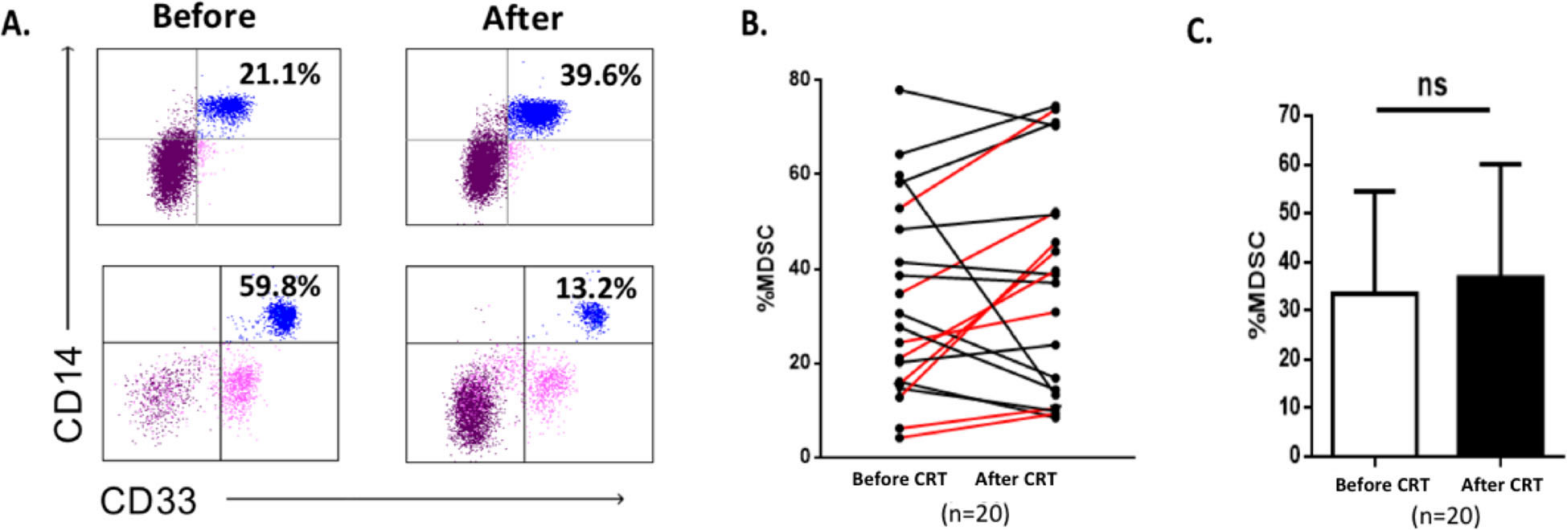
Circulating MDSC cells before and after CRT. PBMCs from 20 patients treated with CRT for lung or head and neck cancer were taken before CRT and 1 month after. HLA-DR^low^ Lineage^−^ CD33^+^CD14^+^CD11b^+^ MDSC were analyzed by flow cytometry. **A** and **D** Representative plots for MDSC (**A**) in two patients. **B** MDSC rates variation after CRT, lines in red representing significant increase (> 20%) from baseline (*n* = 20). **C** MDSC rates before and after CRT (*n* = 20). Results are shown as mean (standard deviation). ns, not significant (Wilcoxon test)

The impact of CRT on Treg and MDSC was evaluated in 20 patients by flow cytometry from PBMCs collected before CRT and 1 month after. We assessed the percentages of circulating myeloid-derived suppressor cells (MDSC) and regulatory T cells (Treg) in viable PBMCs using flow cytometry (Supplementary Figure S1). MDSC were defined as HLA-DR^low^Lin^−^ CD33^+^CD14^+^CD11b^+^ and Treg were defined as CD3^+^CD4^+^CD25^+^CD127^low^FoxP3^+^. A significant increase in Treg was observed in 12 out of 20 patients (60%) after CRT (Fig. 1A and B). The mean Treg rate was 2.7% before CRT and 4.9% after CRT (*p* = 0.0015) (Fig. 1C). Furthermore, the rate of CTLA4^+^ Treg significantly increased after CRT (*p* = 0.003) (Fig. 1D-F). Next, we evaluated the effect of CRT on MDSC and found that overall MDSC rate increased significantly in 8 out of 20 patients (40%) after CRT (Fig. 2A and B). Although the mean MDSC rate increased after CRT, the difference was not statistically significant (Fig. 2C). Altogether, these results suggest that CRT promotes high Treg expansion in the peripheral blood.

### Decrease of peripheral anti-telomerase CD4 Th1 response after CRT

**Fig. 3:**
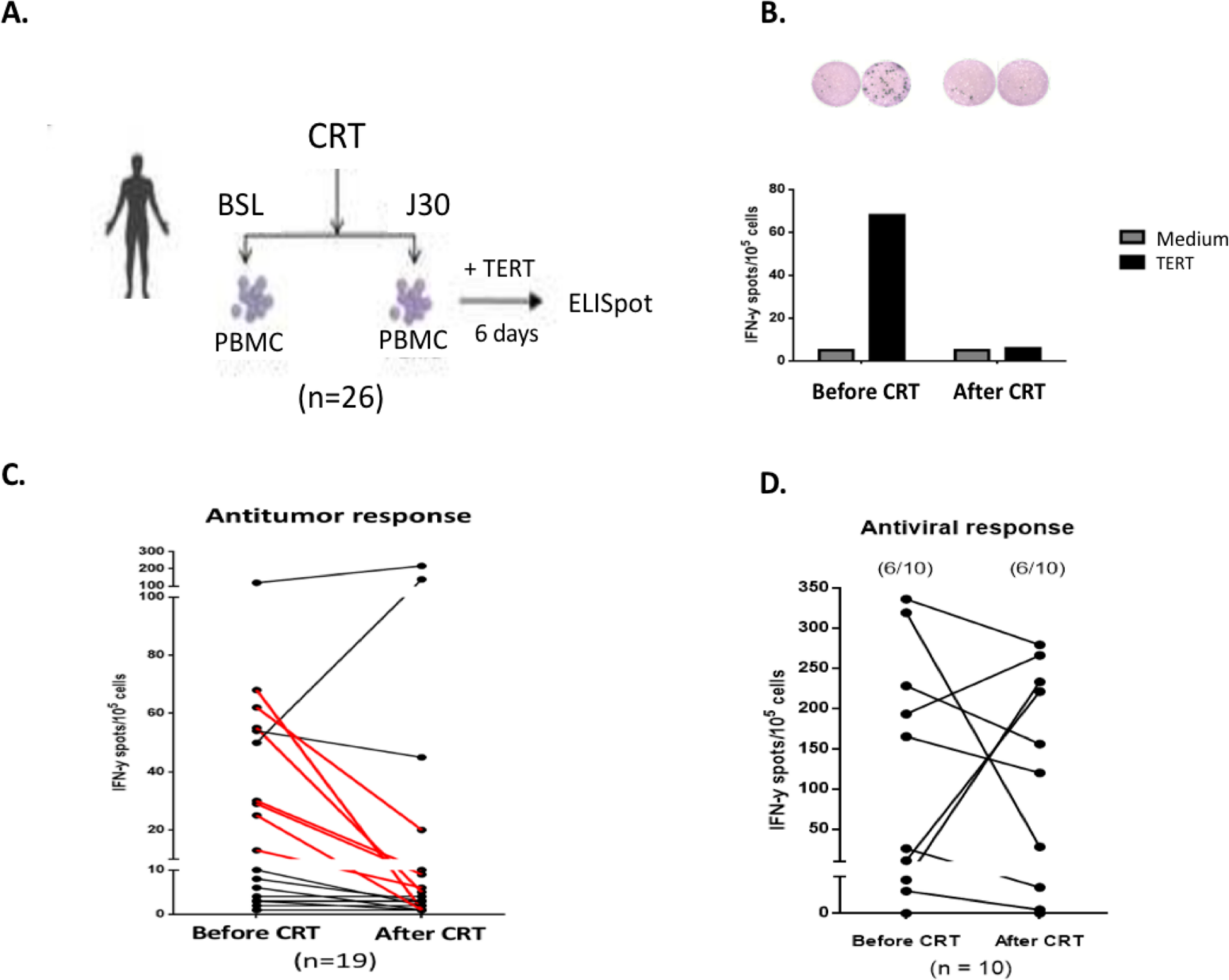
Spontaneous anti-tumor and antiviral responses before and after CRT. **A** PBMCs from 26 patients treated with CRT for a lung cancer or a head and neck cancer were collected before CRT and 1 month after. After short stimulation (1 week) with a mixture of HLA class II peptides derived from TERT or viral peptides, the presence of TERT-specific T cells was detected by IFNγ ELISPOT assay. The results represented specific IFNγ spots after subtraction of background. Responses were positive when IFNγ spots were more than 10 and more than 2-fold the background. **B** Response against TERT in a representative patient with loss of anti-TERT Th1 reponse. Bottom: histograms represented specific IFNγ spots number in medium (grey) and TERT (black). Top: illustration of medium and TERT ELISPOT wells. **C** individual variation of the intensity of anti-TERT Th1 response in patients with available data at baseline and 1 month after CRT (*n* = 19). Lines in red represent significant decrease (> 20%) from baseline. **D** intensity of specific anti-viral response in 10 patients with available data at baseline. Number of patients with anti-TERT response is shown between brackets

We further evaluated the impact of CRT on tumor-specific T cell response. To this end, we measured by IFN-γ ELISpot the CD4 T cell response directed against telomerase (TERT), a shared-tumor antigen (Fig. 3A) [36]. We previously showed that circulating anti-TERT CD4 T cell response is a surrogate marker of the host’s antitumor Th1 immunity and that the presence or induction of circulating anti-TERT CD4 T cell response was associated with a good prognosis in several cancers such as renal carcinoma, anal carcinoma, and NSCLC [33–35, 37, 38]. T cell responses against viruses such as CMV, EBV and FLU measured concurrently were used as control. Anti-TERT Th1 response was found in 12 out of 26 patients (46%) at baseline. Data was missing in three patients. Response against TERT in a representative patient with loss of anti-TERT Th1 response after CRT is shown in Fig. 3B. In 19 patients with available samples before and after CRT, we found that 10/19 had anti-TERT Th1 response at baseline, among which seven (7/10) had a significant decrease of their response, whereas only two patients (2/10) had a significant increase of their anti-TERT Th1 response after CRT (Fig. 3C). In contrast, no obvious change of the frequency or intensity of antiviral recall responses was observed after CRT (Fig. 3D). These results suggest that CRT may induce a decrease of tumor-specific T cells in peripheral blood. Thus, we assessed whether there is a relationship between the decrease of anti-TERT T response and the increase of immunosuppressive cells. Overall, 17 patients had available data for both anti-TERT response and immunosuppressive cells (Table 2). In patients with a decrease in anti-TERT response (6/17) or no response (8/17), we found 10/14 patients (71%) who had an increase in Treg and/or MDSC rates in peripheral blood. Two patients with an increase in anti-TERT response had an increase in Treg rates as well.

**Table 2:**
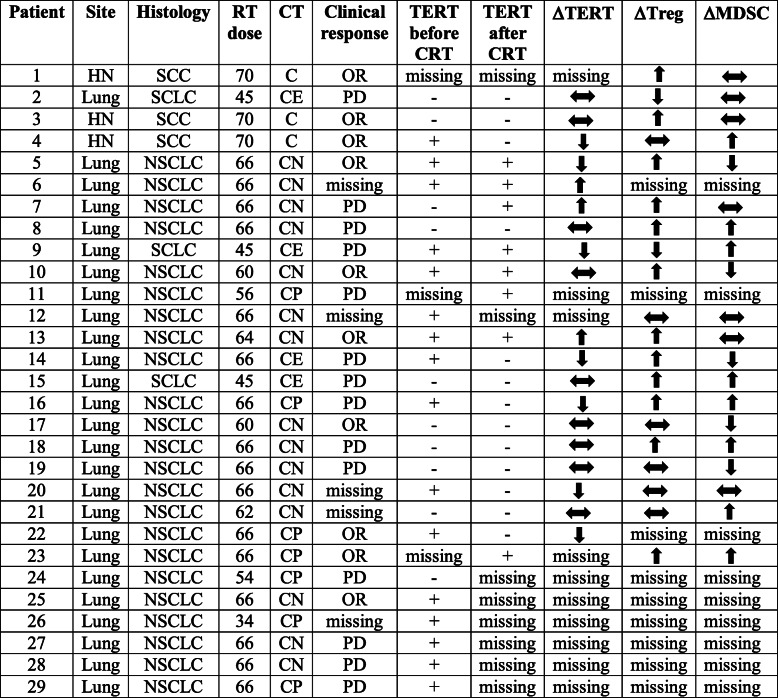
Clinical response, anti-TERT response and immunosuppressive cells in all patients (*n* = 29)

*Abbreviations*: *HN* Head & neck cancer, *SCC* Squamous cell carcinoma, *NSCLC* Non-small cell lung cancer, *SCLC* Small cell lung cancer, *RT* Radiotherapy, *CT* Chemotherapy, *OR* Objective response, *PD* Progressive disease, *TERT* Antitumor response, *C* Cisplatin, *CE* Cisplatin + etoposide, *CN* Cisplatin + navelbine, *CP* Carboplatin + paclitaxel. Δ: evolution of anti-TERT response, Treg and MDSC rates after CRT, defined as stability (), increase () or decrease (). (+) sign represents presence of anti-TERT response, (−) sign represents absence of anti-TERT response

### Influence of CRT-related immune response on clinical response

After a median follow-up of 12 months (range, 3–30 months), the median overall survival and progression-free survival were 28 and 17 months, respectively, similar to previously reported outcomes after CRT in these cancers [39–41]. The clinical response was assessed in 24 patients. The objective response (OR) rate was 10/24 (42%). Progressive disease (PD) was seen in 14 patients (58%).

Next we studied the association of naturally occurring anti-TERT immune response and clinical outcome (Table 2). We found a high frequency of anti-TERT Th1 response among the majority of CRT-responders, compared to non-responders. Indeed, the anti-TERT response was found either at baseline or after CRT in 5/7 patients (71%) with OR, while 4/9 patients (44%) with PD exhibited peripheral anti-TERT CD4 T cell response either at baseline or after CRT. Therefore, patients who were able to mount specific anti-tumor T-cell responses were probably more likely to respond to treatment. Furthermore, we evaluated overall survival according to TERT responses and immunosuppressive cells (Supplementary Figure S2). The median value of TERT response, MDSC and Treg rates was used as a cut-off for TERT low/high, MDSC low/high, and Treg low/high, respectively. There was no significant difference in patients with TERT low or high response (Supplementary Figure S2A), nor between MDSC low or high (Supplementary Figure S2B), and Treg low and high levels (Supplementary Figure S2C) measured before and after CRT.

Next, we evaluated the relationship between clinical response and immunosuppressive cells. There was a significant increase in Treg and/or MDSC in 7/8 patients (88%) with OR, and 7/9 (78%) patients with PD (Table 2). Thus, there was no difference between responders and non-responders with regard to immunosuppressive cells.

Our results suggest that the clinical response was mostly influenced by the peripheral anti-TERT CD4 T cell response and not by immunosuppressive cells.

## Discussion

In this study, we wanted to determine the impact of CRT on anti-tumor specific responses in cancer patients. To this end, we assessed T-cell responses directed specifically against TERT, known for its frequent expression in various cancer types and its high immunogenicity [42]. In our cohort of patients presenting predominantly with a non-small cell lung cancer (NSCLC), we found that T-cell responses against TERT were naturally present in 46% of the cases. This was in line with previous results showing TERT-specific CD4 T cell responses in 45% of patients with non-metastatic NSCLC at baseline [33]. The prognostic value of specific immune responses in the peripheral blood of cancer patients have been reported in several malignancies. For instance, Masterson et al. demonstrated that the presence of E7-specific immune responses in the peripheral blood of HPV^+^ head and neck squamous cell carcinoma patients was associated with better overall survival [43]. Interestingly, our results suggested that patients who were able to mount specific anti-tumor T-cell responses were more likely to respond to treatment. However, we demonstrated a significant decrease of anti-TERT responses after CRT in most of the patients. The loss of anti-tumor specific immune responses could not be related to a global T-cell anergy, as illustrated by the unchanged antiviral recall responses’ frequency. We hypothesized that the decrease of tumor-specific-T cell responses after CRT was mainly related to RT by promoting suppressive cells’ expansion.

Indeed, evidence support the ambivalent role of RT in activating the host antitumor immunity while promoting immunosuppression [3, 9, 21]. The induction of suppressive Treg and MDSCs after CRT has been previously reported. Hence, Schuler et al. reported the amplification of highly suppressive, cisplatin-resistant Treg after CRT and these cells persist long-term after treatment and could be responsible for suppression of antitumor immune responses and recurrence in HNSCC [44]. Recently, Hanoteau et al. showed that removal of MDSC in vivo improves CRT effectiveness [45]. Furthermore, [46] studied the impact of RT and CRT in patients with cervical cancer and showed that RT alone or with concurrent CT led to a decrease in T-cell activation. Santin et al. showed that RT and cisplatin-based CRT decreased Phytohemagglutinin (PHA)-induced T-cell proliferation and lymphocytes count in locally advanced cervical cancer [47]. Here, we observed a significant expansion of circulating Treg and MDSC after CRT. Although, the suppressive functions of these cells were not formally explored, we speculated that these cells could be involved in the decrease of antitumor Th1 response observed after CRT.

Cisplatin or carboplatin-based CT was commonly used in combination with RT, both in head and neck and lung cancer. These drugs have been shown to stimulate host antitumor immunity either by increasing tumor cells sensibility to immune effector cells attack or through elimination of immune suppressive cells [48, 49]. In line with this, we previously reported that cisplatin-based CT reinvigorates TERT-specific Th1 response by promoting MDSC depletion [35, 37, 50]. Thus, our data also suggest that the inhibitory effect of RT rather than platinum-based CT was responsible of the attenuation of tumor-specific T cell responses.

Our study has several limitations. First, our analyses were limited to peripheral immunity rather than the tumor microenvironment. Second, the relatively small number of patients included makes it difficult to perform robust statistical analysis. Third, analyses were performed before and after CRT without providing an interim analysis during treatment which could have allowed the understanding of CRT’s early impact on anti-tumor immune responses. Currently, we are recruiting patients treated with CRT for locally advanced inoperable lung or head and neck cancer to study the mechanisms underlying the immunomodulation induced by CRT in a prospective large cohort (iRTCT cohort, NCT 03117946).

## Conclusion

This study emphasized the role of CRT in the modulation of systemic immune responses. We found that after CRT there was a decrease in anti-TERT response in most of the patients that could be explained by the concomitant increase in immunosuppressive cells, which was predictive of the clinical response. These preliminary results have implications in clinical practice particularly in combination strategy with immune checkpoint inhibitors.

## Material and methods

### Patients and blood samples

Lung cancer patients and head and neck cancer patients treated with CRT at the department of radiation oncology of the University Hospital of Besancon (France) were enrolled. All patients were included after the signature of informed consent, in accordance with the French regulation and after approval by the local ethics committee. Blood samples were collected prior to treatment and 1 month after. Peripheral Blood Mononuclear Cells (PBMCs) were Ficoll-isolated (Amersham, Biosciences, France) and frozen in aliquots in liquid nitrogen. Approximately 30 ml of blood were collected before and after treatment (1 month later). This allowed the isolation of 15–20 × 10^6^ PBMCs at baseline and around 10^7^ PBMCs 1 month after CRT. After thawing, cell viability was estimated around 90%.

Clinical response to treatment was evaluated 3 months after the end of CRT with CT scan based on RECIST criteria. Objective response was defined as a complete response, a partial response or a stable disease. Otherwise, progressive disease was stated. Patients with progressive disease after CRT have been treated according to the standard of care. In this limited cohort, no patient received adjuvant immunotherapy (eg, Durvalumab) following CRT at the time of the study.

### Synthetic peptides

To measure telomerase-specific CD4 Th1 responses in peripheral blood, we used a mixture of eight highly promiscuous telomerase-derived 15 mer HLA-DR-binding peptides (referred to as UCP1, UCP2, UCP3 and UCP4) and HLA-DP4-binding 15-mer peptides (p541–55, p573–84, p613–27 and p911–25) previously described by our group [36, 38, 50]. These peptides bind to most prevalent HLA class II molecules which increases their use to a large number of cancer patients. To evaluate the antiviral T-cell responses, we used peptide mixtures derived from influenza virus (Flu), Epstein Barr virus (EBV), and cytomegalovirus (CMV) (PA-CEF-001), which were purchased from JPT (Germany) or CTL (Cellular Technology Ltd., Germany) at > 80% purity.

### In vitro stimulation for the detection of tumor-specific CD4+ Th1 responses in blood

Telomerase-specific CD4+ T-cell responses were assessed in PBMCs using a standard IFNγ ELISPOT assay, following in vitro stimulation. Briefly, PBMCs (3.10^6^ cells per well) were cultured for 6 days in a 24-well plate in RPMI containing 5% human serum and 1% penicillin-streptomycin, along with the mixture of TERT-derived peptides (5 μg/mL). To assess anti-viral T cell responses, cells were stimulated with a mixture of peptides derived from CMV, EBV and Flu (1 μg/mL). Recombinant interleukine (IL) 7 (5 ng/mL, R&D Systems, France) was added on day 1, and recombinant IL-2 (50 U/mL, Proleukin, Chiron, France) was added on day 3. Plates were incubated at 37 °C.

### IFNγ ELISPOT assay

The presence of peptide-specific T cells was measured by IFNγ ELISpot assay at day 7 according to the manufacturer’s instructions (Diaclone, France), as previously reported [36]. Briefly, lymphocytes from in vitro stimulation were incubated for 17 h at 37 °C in duplicates or triplicates (10^5^ per well) in a precoated 96-well ELISpot plate with anti-human IFNγ monoclonal antibody, with 5 μg/mL of the peptide mixtures derived from TERT and CEF in the X-vivo 15 medium (Lonza). Cells cultured with medium alone or phorbol myristate acetate (PMA, 100 ng/mL; Sigma-Aldrich) and ionomycin (10 μmol/L; Sigma-Aldrich) were used as negative and positive controls, respectively. The IFNγ spots were revealed following the manufacturer’s instructions (Diaclone, 856051020P). IFNγ secreting cells i.e., spot-forming cells were counted using the C.T.L. Immunospot system (Cellular Technology Ltd). After subtracting the negative control values (background), we calculated the number of IFNγ spots per 10^5^ cells. A response was considered positive if the number of IFNγ spots per 10^5^ cells was > 10 and more than two times the background.

### Analysis of circulating immunosuppressive cells by flow cytometry

To discriminate live from dead cells, PBMCs were first washed in 1× PBS (Gibco) and stained with Fixable viability dye (FvD)-eFluor 506 according to the manufacturer’s instructions. For MDSC analysis, 10^6^ cells were surface-stained in the dark for 30 min at 4 °C with a mixture of the following antibodies: PerCP-Cy5.5 anti-human HLA-DR, BV421 anti-human CD14, APC anti-human CD33, and PE-Cy7 anti-human CD11b. Lineage cocktail (Lin-) was composed of anti-human CD19 APC Alexa Fluor 750, CD56 APC Alexa Fluor 750, and CD3 APC Alexa Fluor 750. The following isotype controls were used for anti-CD11b: PE-Cy7 mouse IgG1, and for anti-CD33: APC mouse IgG1.

For Treg analysis, 10^6^ cells were first stained with surface antibodies against: CD3-APC, CD4-alexa488, CD25-BV421 Pacific Blue, and CD127-PerCP-Cy5.5. Intracellular staining was performed following the manufacturer’s instructions (Becton Dikinson biosciences). Cells were fixed and permeabilized with Human FoxP3 buffer set and then stained with antibodies against FoxP3-APC (clone 259D/C7; Biolegend) and CTLA4-PE (clone BNI3; Becton Dikinson).

All antibodies used are referenced in Supplementary Table S1. The stained samples were acquired on a FACS CantoII cytometer and analyzed with Diva software (Franklin Lakes, NJ, USA). Around 100,000 events in viable cells were measured for each sample. According to our previous works, an increase or decrease of 20% of Treg or MDSC rate after treatment was considered as significant [34, 35, 51].

### Statistical analysis

Data are presented using mean values +/− standard deviation (SD). Statistical comparison between groups was based on Wilcoxon test using Prism 6 GraphPad Software. Survival curves were calculated with the Kaplan–Meier method. A *p* value ≤0.05 was used as the cutoff for significance.

## Supplementary Information

**Additional file 1: Supplementary Figure S1.** Gating strategy for flow cytometry analyses. The figure shows the gating strategy to Treg (**A**) and MDSC (**B**) populations. Frequencies of Treg cells were observed in CD4 T-cell population. Expression of CD127, FoxP3, and CTLA4 were analyzed on Treg (**A**). MDSC populations were analyzed after exclusion of lineage (CD3, CD56, CD19)(**B**).

**Additional file 2: Supplementary Figure S2.** Kaplan-Meier overall survival (OS) curves in patients according to TERT specific T-cell responses and immunosuppressive cells levels before and after CRT. OS according toTERT-specific responses levels **(A),** MDSC levels **(B),** andTreg levels **(C)**.

**Additional file 3: Supplementary Table S1.** List of monoclonal antibodies used for flow cytometry.

## Data Availability

The datasets used and/or analysed during the current study are available from the corresponding author on reasonable request.
