# Supplementary material for: Cisplatin-based chemoradiation decreases telomerase-specific CD4 TH1 response but increases immune suppressive cells in peripheral blood
Source: BMC Immunol. 2021 Jun 18;22:38. doi: 10.1186/s12865-021-00429-5 (PMC8212531; doi:10.1186/s12865-021-00429-5)
Supplement: Supplementary file 1 — Additional file 1: Supplementary Figure S1. Gating strategy for flow cytometry analyses. The figure shows the gating strategy to Treg (A) and MDSC (B) populations. Frequencies of Treg cells were observed in CD4 T-cell population. Expression of CD127, FoxP3, and CTLA4 were analyzed on Treg (A). MDSC populations were analyzed after exclusion of lineage (CD3, CD56, CD19)(B). [file 12865_2021_429_MOESM1_ESM.pdf]

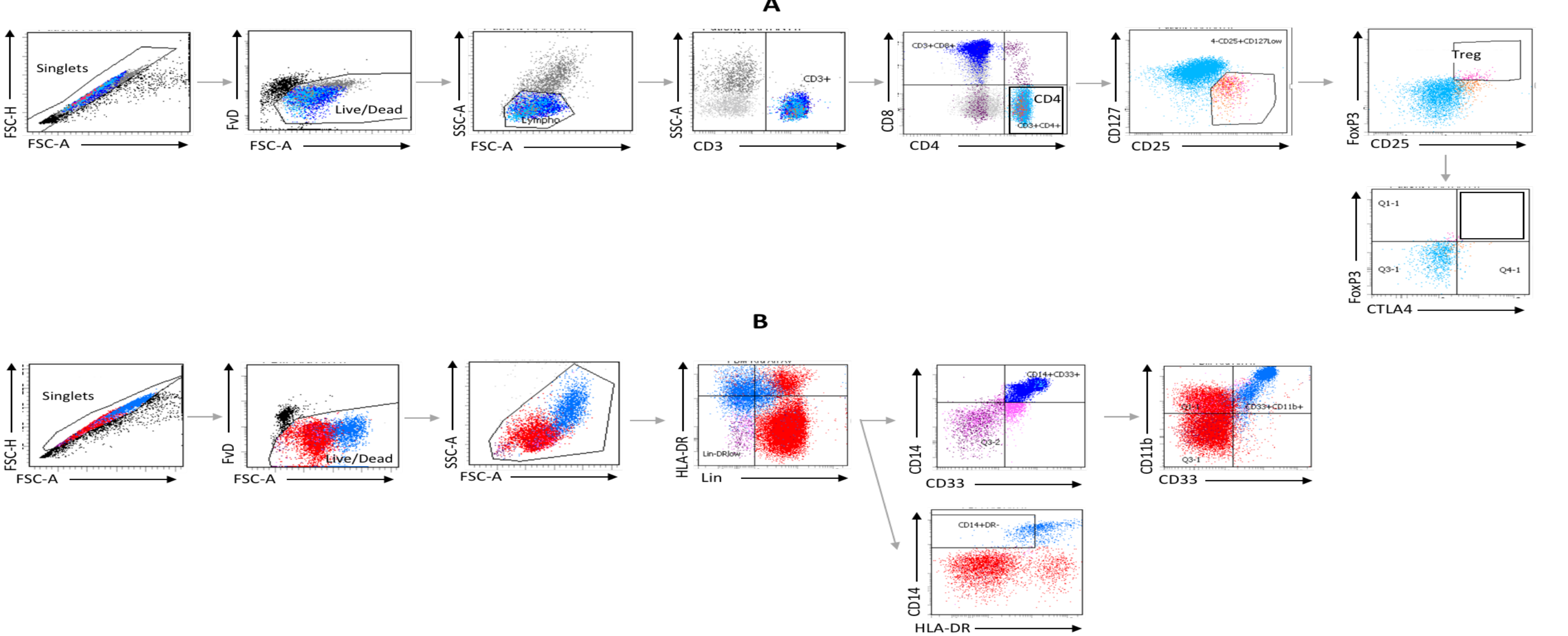

Figure S1. Gating strategy for flow cytometry analyses. The figure shows the gating strategy to Treg (**A**) and MDSC (**B**) populations. Frequencies of Treg cells were observed in CD4 T-cell population. Expression of CD127, FoxP3, and CTLA4 were analyzed on Treg (**A**). MDSC populations were analyzed after exclusion of lineage (CD3, CD56, CD19)(**B**).
