# Supplementary material for: Cisplatin-based chemoradiation decreases telomerase-specific CD4 TH1 response but increases immune suppressive cells in peripheral blood
Source: BMC Immunol. 2021 Jun 18;22:38. doi: 10.1186/s12865-021-00429-5 (PMC8212531; doi:10.1186/s12865-021-00429-5)
Supplement: Supplementary file 2 — Additional file 2: Supplementary Figure S2. Kaplan-Meier overall survival (OS) curves in patients according to TERT specific T-cell responses and immunosuppressive cells levels before and after CRT. OS according toTERT-specific responses levels (A), MDSC levels (B), andTreg levels (C). [file 12865_2021_429_MOESM2_ESM.pdf]

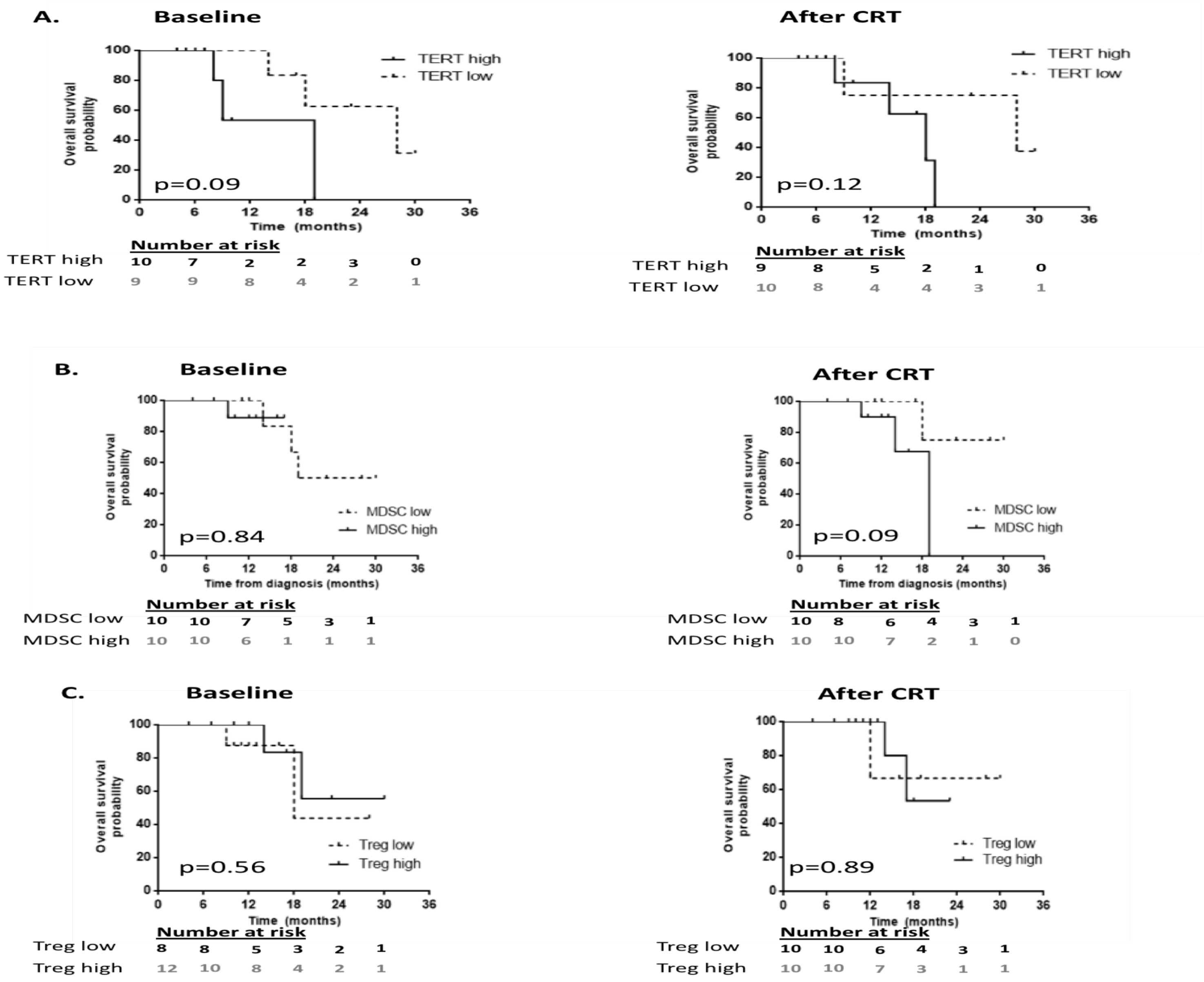

Supplementary Figure S2. Kaplan-Meier overall survival (OS) curves in patients according to TERT specific T-cell responses and immunosuppressive cells levels before and after CRT. OS according toTERT-specific responses levels **(A),** MDSC levels (**B),** Treg levels **(C).**
