# Supplementary material for: Cisplatin-based chemoradiation decreases telomerase-specific CD4 TH1 response but increases immune suppressive cells in peripheral blood
Source: BMC Immunol. 2021 Jun 18;22:38. doi: 10.1186/s12865-021-00429-5 (PMC8212531; doi:10.1186/s12865-021-00429-5)
Supplement: Supplementary file 3 — Additional file 3: Supplementary Table S1. List of monoclonal antibodies used for flow cytometry. [file 12865_2021_429_MOESM3_ESM.docx]

**Supplementary Table S1.** List of monoclonal antibodies used for flow cytometry

| ANTIBODIES | SOURCE | Fluorochromes |
| --- | --- | --- |
| anti human CD19  (clone J3-119) | Beckman Coulter | APC alexa750 |
| anti human CD56  (clone N901) | Beckman Coulter | APC alexa750 |
| anti human CD3  (clone A94680) | Beckman Coulter | APC alexa750 |
| anti human CD11b  (clone ICRF44) | Becton Dickinson | PE Cy7 |
| anti human CD33  (clone WM53) | Becton Dickinson | APC |
| anti-mouse IgG1 isotype control (clone MOPC-21) | Becton Dickinson | PE Cy7 |
| anti-mouse IgG1 isotype control  (clone MOPC-1) | Becton Dickinson | APC |
| anti human HLA DR  (clone L243) | Biolegend | PerCP Cy5.5 |
| anti human CD14  (clone HCD14) | Biolegend | BV421 Pacific Blue |
| anti human CD3  (clone UCHT1) | Biolegend | APC Fire 750 |
| Fixable Viability Dye | eBiosciences | eFluor506 |
| anti human CD4  (clone RPA-T4) | Biolegend | alexa488 |
| anti human CD8  (clone SK1) | Biolegend | PE-Cy7 |
| anti-human CD3  (clone UCHT1) | Becton Dickinson | APC Fire750 |
| anti-human CD25  (clone M-A251) | Becton Dickinson | BV421/Pacific Blue |
| anti-human CD127  (clone eBioRDR5) | eBioscience | PerCP-Cy5.5 |
| anti-human FoxP3  (clone 259D) | Biolegend | APC/alexa 647 |
| anti-human CTLA-4  (clone BNI3) | Becton Dickinson | PE |
